# Long non-coding RNA MEG3 inhibits neovascularization in diabetic retinopathy by regulating microRNA miR-6720-5p and cytochrome B5 reductase 2

**DOI:** 10.1080/21655979.2021.2000721

**Published:** 2021-12-07

**Authors:** Jinpeng Chen, Lin Liao, Huiyong Xu, Zheng Zhang, Jian Zhang

**Affiliations:** a Department of Ophthalmology, Ezhou Central Hospital, Ezhou, China; b Department of Ophthalmology, Wuhan Fourth Hospital, Wuhan, China

**Keywords:** LncRNA MEG3, diabetic retinopathy, proliferation, migration, neovascularization

## Abstract

Diabetic retinopathy (DR) is a major cause of vision loss in working and elderly populations. long non-coding RNA (LncRNA) MEG3 is thought to have some effect on DR, but the exact mechanism remains to be clarified. The expression levels of lncRNA MEG3, miR-6720-5p, and cytochrome B5 reductase 2 (CYB5R2) in human retinal microvascular endothelial cells (hRMECs) were detected by quantitative reverse transcription polymerase chain reaction (qRT-PCR). 3-(4,5-Dimethylthiazol-2-yl)-2,5-diphenyltetrazolium bromide (MTT), transwell migration, and tube formation assays were used to determine the cell viability, migration, and tube formation ability of hRMECs, respectively. The interaction of MEG3, miR-6720-5p, and CYB5R2 was detected and explored by a luciferase assay. The expression of MEG3 and CYB5R2 was upregulated and that of miR-6720-5p was downregulated in patients with DR and hRMECs treated with high glucose. Knocking down MEG3 or CYB5R2 promoted proliferation, migration, and neovascularization in hRMECs. The intervention of miR-6720-5p reversed the effect of MEG3 knockdown on hRMEC function, and this effect was eliminated by silencing CYB5R2. Therefore, MEG3 acted as a sponge to suppress miR-6720-5p and regulate the expression of CYB5R2, thereby inhibiting DR neovascularization.

## Introduction

Diabetic retinopathy (DR) is a specific microvascular complication of diabetes and is the leading cause of vision loss among adult workers and the elderly [1]. It has been reported that about one-third of diabetic patients suffer from DR [2]. Moreover, a meta-analysis of population data from developed countries estimated that more than 90 million people suffer from DR [3]. Severe DR leads to a decline in the quality of life of patients, and the patients require a considerable amount of medical resources, resulting in an increased economic burden on countries [4,5]. Therefore, there is an urgent need to develop an effective DR treatment strategy.

Long non-coding RNA (LncRNA) have been identified as transcripts of > 200 nucleotides and are involved in a variety of biological processes, such as cell proliferation, migration, cell cycle, apoptosis, and angiogenesis [6]. Previous studies have found that the dysregulation of lncRNA expression is often associated with complex human diseases involving vascular biology [7]. In addition, in recent years, there has been increasing evidence that lncRNAs may play a role in suppressing or worsening DR by modulating gene expression at transcriptional, post-transcriptional, or epigenetic levels [8]. For example, H19, which regulates the endothelial cell transformation in DR, was downregulated in the vitreous of DR patients and high glucose (HG)-induced endothelial cells [9]. LncRNA BANCR is poorly expressed in patients with DR; however, its overexpression may be a novel therapeutic approach of DR through inhibition of apoptosis of ARPE-19E-19 cells under HG conditions [10]. The lncRNA MEG3 has a therapeutic effect on DR in rats as it improves the retinal architecture [11]. Furthermore, the lncRNA MEG3 was expressed at low levels in the serum of patients with DR, while its overexpression inhibited the occurrence of DR through the in vitro inhibition of vascular endothelial growth factor (VEGF) in ARPE-19 cells treated with HG [12]. Although lncRNA MEG3 has a certain efficacy in the treatment of neovascularization in DR, its regulatory mechanism has not been fully explored.

MicroRNAs (miRNAs), approximately 22 nucleotides in length, induce mRNA instability or inhibit protein translation through the regulation of transcription or post-transcription gene expression [13]. As an emerging miRNA, miR-6720-5p has not been thoroughly studied. Sporadic reports have found that miR-6720 is expressed at low levels in gliomas [14]. MiR-6720 may also influence the prognosis of papillary thyroid cancer through the hedgehog and calcium pathways [15]. However, whether MEG3 participates in DR neovascularization through the regulation of miR-6720-5p remains unclear. Furthermore, to the best of our knowledge, no downstream target mRNA of miR-6720-5p has been reported till date. In this study, the downstream target of miR-6720-5p in DR was discussed for the first time based on the targeting effect of miRNA on mRNA, which is another novel point of interest.

CYB5R2, a flavoprotein, is involved in many oxidation and reduction reactions [16]. It has been reported that CYB5R2 regulates angiogenesis-related genes, thereby reducing angiogenesis around tumors [17]. In DR, only one study revealed the downregulation of CYB5R2 through a multi-bioinformatics analysis based on integrated gene expression profile data; however, its mechanism and function have not been fully explored [18]. Therefore, this study investigated the role of CYB5R2 in DR neovascularization.

We hypothesized that MEG3 might play a potential role in DR angiogenesis through certain regulatory mechanisms. In the present study, we investigated the expression of MEG3, miR-6720-5p, and CYB5R2 in DR plasma and retinal tissue, and HG-induced human retinal microvascular endothelial cells (hRMECs). Additionally, the effects of MEG3/miR-6720-5p/CYB5R2 on the proliferation, apoptosis, migration, and neovascularization of human hRMECs were evaluated. The aim of this study was to provide a potential target for the clinical treatment of DR neovascularization.

## Methods

### Clinical samples

Patients with DR (N = 30), diabetic patients without retinopathy (NDR = 30), and healthy subjects as control (HC, n = 30) were enrolled in this study. There were no significant differences in age or sex among the three groups. This study was approved by the Ethics Committee of the Ezhou Central Hospital. Informed consent was obtained from all the enrolled patients and healthy subjects. About 5 mL of blood was withdrawn from all subjects after they had fasted for 8 h; the blood sample was centrifuged at 4℃ at 1200 × g for 10 min to prepare the plasma sample, which was stored at −80℃. The clinical and biochemical characteristics of the study subjects are shown in Table 1.

**Table 1.** Comparisons of baseline characteristic of subjects among the DR, NDR and control groups

Indicators	Control	NDR	DR
Gender (male/female)	17/13	18/12	16/14
Age (years)	46.39 ± 7.83	45.73 ± 8.33	48.61 ± 7.19
Course of disease (years)	/	8.15 ± 2.46	8.47 ± 2.92
BMI (kg/m^2^)	23.96 ± 1.36	22.71 ± 1.82	23.41 ± 1.09
FBG (mmol/L)	4.04 ± 0.79	6.94 ± 2.15*	12.06 ± 2.27^*#^
HbA1c (%)	4.12 ± 0.58	7.82 ± 2.24*	12.73 ± 1.47^*#^
TC (mmol/L)	3.52 ± 0.37	6.04 ± 0.82*	4.38 ± 1.51*
HDL-C (mmol/L)	1.56 ± 0.43	1.35 ± 0.57	1.72 ± 0.64*
LDL-C (mmol/L)	1.89 ± 0.74	2.12 ± 1.33*	2.03 ± 1.25*
FPG (mmol/L)	5.27 ± 1.97	9.61 ± 2.46*	10.46 ± 2.32^*#^
TG (mmol/L)	1.38 ± 0.41	4.41 ± 0.98*	4.63 ± 1.15*
BUN (mmol/L)	4.62 ± 0.73	7.94 ± 0.53*	8.02 ± 0.49*
FIns (pmol/mL)	3.04 ± 1.71	4.78 ± 2.56*	4.99 ± 1.04*
Cr (mmol/d)	52.77 ± 7.64	68.83 ± 1.76*	69.32 ± 1.37*
IL-1 (ng/L)	22.92 ± 7.15	29.55 ± 6.78*	35.22 ± 7.94^*#^
IL-6 (ng/L)	95.74 ± 20.64	201.82 ± 27.69*	227.19 ± 46.26^*#^
TNF-α (ng/L)	7.83 ± 1.96	20.15 ± 3.38*	20.97 ± 3.04*
VEGF (ng/L)	78.65 ± 7.38	93.57 ± 9.45*	109.25 ± 13.66^*#^

Note: DR patients; NDR, non-DR diabetic patients; DR, diabetic retinopathy; FBG, fasting blood glucose; FPG, fasting plasma glucose; TG, triglyceride; TC, total cholesterol; HbA1c, glycosylated hemoglobin; BUN, blood urea nitrogen; Cr, creatinine; HDL-C, high-density lipoprotein cholesterol; LDL-C low-density lipoprotein cholesterol; FIns, fasting insulin; IL, interleukin; TNF, tumor necrosis factor; VEGF, vascular endothelial growth factor.

### Cell culture and treatment

hRMECs were purchased from American Type Culture Collection (Manassas, VA, USA), and were cultured in endothelial cell medium (Gibco, Waltham, MA, USA) containing 10% fetal bovine serum (FBS; Gibco) and 1% penicillin-streptomycin. The cultures were then maintained in an incubator at 37°C with 5% CO_2_. hRMECs were treated with 5.5 mM glucose (NG), 5.5 mM glucose, 19.5 mM mannitol (OS), and 25 mM glucose (HG) to examine the effect of glucose on cells.

### Animal model

Animal experiments were performed in accordance with the guidelines of the Animal Care and Use Committee of our hospital. Male Sprague-Dawley rats (200 ± 20 g; 8-weeks-old) were obtained from Hunan SJA Laboratory Animal Company Co. Ltd. (China), and were placed in a room with humidity of 55% ± 10, a 12 h of light /12 h of dark cycle, and temperature of 22°C ± 1, with free access to food and water. Diabetes was induced in mice through an intraperitoneal injection of streptozotocin (STZ) (65 mg/kg citrate buffer (pH 4.5)) one week after acclimation to the new environment. The control rats received the same volume of citrate buffer with no STZ. Rats with blood glucose over 16.7 mmol/L between 72 h and 1 week of STZ treatment were identified as diabetic models [19]. After eight weeks of induced diabetes, the mice were sacrificed, and the retinal tissue was collected.

### Glucose analysis

The rats were intraperitoneally injected with glucose (Sigma-Aldrich, St. Louis, MO, USA) at a dose of 2.0 mg/g body weight, with subsequent fasting for 16 h before glucose detection. Blood glucose levels were measured using a portable glucose meter (Lifescan, Johnson & Johnson, Milpitas, CA, USA) [20].

### Quantitative reverse transcription polymerase chain reaction (qRT-PCR)

Total RNA was extracted with the Norgen Biotek Total RNA Purification Kit (PA, USA) and cDNA samples were prepared using the SuperScript IV Reverse Transcription Kit (Thermo Fisher Scientific, Waltham, MA, USA). The detection of mRNA by qRT-PCR was performed on an IQ5 thermal cycler (Bio-Rad, Hercules, CA, USA) using IQ SYBR Green Supermix (Bio-Rad). GAPDH was used as a control for MEG3 and CYB5R2.

The Qiagen miRNeasy Serum/Plasma Kit (Qiagen, Hilden, Germany) was used to collect miRNAs in the plasma. MiRNAs were isolated from cells and tissues using a miRNA isolation kit (Ambion, Austin, TX, USA). Reverse transcription of miRNA was performed using the Ncode™ miRNA first-strand cDNA synthesis kit (Invitrogen, Waltham, MA, USA). QRT-PCR was performed using an All-in-One™ miRNA qRT-PCR Detection Kit (GeneCopoeia, Rockville, MD, USA). U6 acted as an endogenous control factor for miR-6720-5p The relative expression of each gene was determined by the 2-^ΔΔC^_T_ method [21]. The sequences of the primers used in this study are listed in Table 2.

**Table 2.** Sequence of the primers used in this study

gene	primer type	Sequence
ACSM3	Forward	5’-AGGAAGATGCTACGTCATGCC-3’
	Reverse	5’-ATCCCCAGTTTGAAGTCCTGT-3’
DSCC1	Forward	5’-CGTGGTGATAAAGACGAGCA-3’
	Reverse	5’-CCGGAGTTTTACAACCAGGA-3’
SARDH	Forward	5’-AGCGACCTGACTGTTGCC-3’
	Reverse	5’-CCTGTAGCACCGTGTTTATG-3’
CYB5R2	Forward	5’-CCTTGTAGGGACCCGTCCC-3’
	Reverse	5’-GACAGGGGTGTAAGCCCTG-3’
EHHADH	Forward	5’-AAACTCAGACCCGGTTGAAGA-3’
	Reverse	5’-TTGCAGAGTCTACGGGATTCT − 3’
miR-494-3p	Forward	5’-AACGAGACGACGACAGAC-3’
	Reverse	5’-TGAAACATACACGGGAAACCTC-3’
MEG3	Forward	5’-CTCCCCTTCTAGCGCTCACG-3’
	Reverse	5’-CTAGCCGCCGTCTATACTACCGGCT-3’
GAPDH	Forward	5’-CAATGACCCCTTCATTGACC-3
	Reverse	5’-GACAAGCTTCCCGTTCTCAG-3’
U6	Forward	5’-AACGAGACGACGACAGAC-3
	Reverse	5’-GCAAATTCGTGAAGCGTTCCATA-3’

### Cell transfection

MEG3 specific siRNA (si-MEG3), CYB5R2 specific siRNA (si-CYB5R2), and a siRNA-negative control (si-NC) were synthesized by System Biosciences (Palo Alto, CA, USA). The miR-6720-5p mimic, miR-6720-5p inhibitor, and corresponding negative controls (mimic-NC and inhibitor-NC) were obtained from Switchgear Genomics (Carlsbad, CA, USA). The Lipofectamine 2000 Transfection Reagent (Invitrogen, Waltham, MA, USA) was used to transfect 100 nM siRNA, 100 nM miR-6720-5p mimic, or 75 nM inhibitor into hRMECs from the HG group.

### 3-(4,5-Dimethylthiazol-2-yl)-2,5-diphenyltetrazolium bromide (MTT) assay

hRMECs that were transfected for 48 h were collected and placed in a 96-well culture plate. The MTT solution (5 mg/mL, Beyotime Biotechnology, Haimen, China,) was added to each well after the cells adhered, and the cells were then incubated for 4 h. The culture medium was discarded, and 100 μL dimethyl sulfoxide was added to each well. Cell absorbance was measured using a microplate reader (Bio-Tek, Winooski, VT, USA) at 490 nm [22].

### Flow cytometry assay

Cell apoptosis was measured using the Propidium Iodide-Annexin V Apoptosis Detection Reagent Kit (KeyGen Tech, USA), as directed by the manufacturer. Briefly, after cell collection and rinsing, the cells were suspended in 500 μL of the combined buffer at a density of 1 × 10^6^ cells/mL. Annexin V (5 mL) and propidium iodide (10 μL; 20 g/L) were then added to the suspension. After incubating at room temperature in the dark for 5 min, the cell apoptosis rate was analyzed using a FACSCalibur flow cytometer (BD Biosciences, East Rutherford, NJ, USA) [23].

### Transwell assay

After transfection for 48 h, the hRMECs were digested and diluted to 5 × 10^4^ cells/mL, and resuspended in a serum-free medium. 200 μL suspension was added to the upper layer of the transwell chamber, and 600 μL of medium containing 20% FBS was added to the lower part of the chamber. The cells were incubated for 24 h and washed with PBS. Subsequently, the cells in the upper chamber wiped off, and the cells in the lower chamber were fixed with 4% paraformaldehyde for 20 min at 25°C and stained with 0.1% crystal violet for 1 h. Photographs were taken using an IX70 microscope (Olympus, Tokyo, Japan), and the number of migratory cells were calculated [24].

### Tube formation assay

Capillary-like structures and the number of hRMECs present were determined by the tube formation assay. After transfection for 48 h, hRMECs were seeded into 48-well plates precoated with Matrigel (Corning, Corning, NY, USA) Each well contained 5 × 10^4^ cells. The culture plate was incubated for 24 h and then photographed under a microscope. The number of tube connections was recorded, and the relative tubulogenesis was calculated [25].

### Luciferase assay

Luciferase reporter analysis was performed as previously reported [26]. The 3’-untranslated region (3’-UTR) of MEG3/CYB5R2 was cloned into the PGL3 vector (Promega, Madison, WI, USA) to obtain wild-type (WT) MEG3/CYB5R2. Mutant (MUT) MEG3/CYB5R2 were obtained using a Quick-change site-directed mutagenesis kit (Stratagene, San Diego, CA, USA). The miR-6720-5p mimic or mimic-NC was transfected into hRMECs with either MEG3-/CYB5R2-WT or MEG3-/CYB5R2-MUT. Relative luciferase activity was detected using a dual luciferase reporter assay system (Promega, Madison, WI, USA) after 48 h of transfection.

### Statistics

Statistical analysis was performed using SPSS (version 17.0, SPSS, Armonk, NY, USA). An unpaired Student’s t-test was used to analyze data between the two groups. One-way analysis of variance (ANOVA) was used to analyze the data between multiple groups. Results are presented as mean ± standard deviation (SD); P values less than 0.05 were considered statistically significant.

## Results

In this study, the regulatory effect of the lncRNA MEG3/miR-6720-5p/CYB5R2 signal axis on neovascularization in DR was investigated. The expression and targeting relationship of these genes were detected in the plasma of DR patients, retinal tissues of DR model rats, and hRMECs induced by HG. In addition, proliferation, apoptosis, and migration of hRMECs as well as degree of angiogenesis were investigated. We hypothesized that MEG3 alleviates the neovascularization of DR, and mechanistically serves as a competitive endogenous RNA (ceRNA) to regulate miR-6720-5p and CYB5R2.

### MEG3/miR-6720-5p/ CYB5R2 axis associated with DR

**Figure 1.**
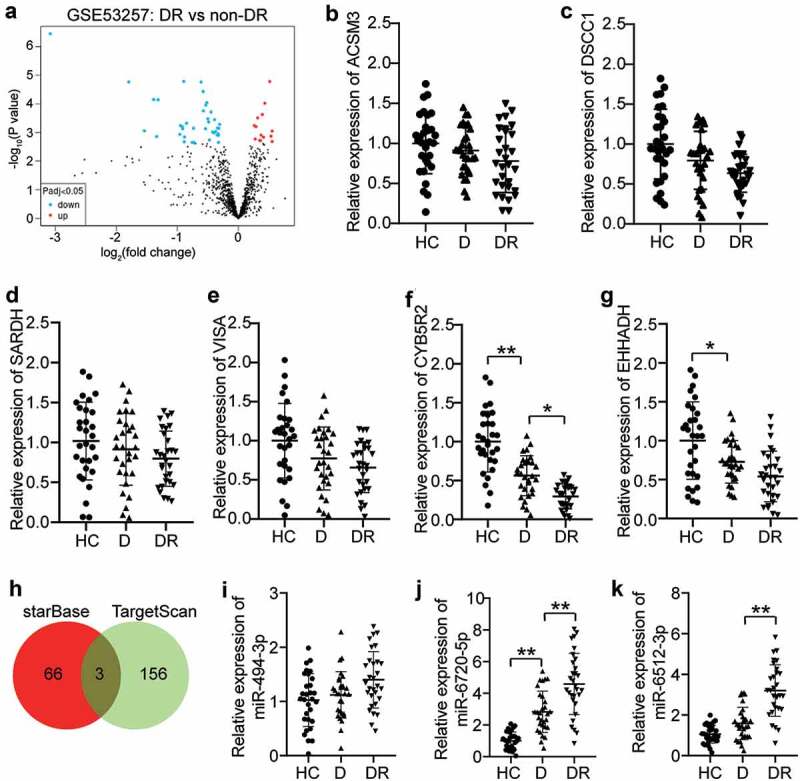
CYB5R2 and miR-6720-5p are associated with DR. (a) The DEGs in GSE53257 with adj.P < 0.05. GSE63257, mRNA expression profile including 6 DR samples and 5 non-DR samples. (b-g) The expression levels of ACSM3, DSCC1, SARDH, VISA, CYB5R2 and EHHADH in blood samples from DR, D and HC patients. DR, diabetic retinopathy. D, diabetes. HC, healthy control. (h) miR-494-3p, miR-6720-5p and miR-6512-3p were the common miRNAs binding to MEG3 and CYB5R2. starBase, an online tool for the prediction of miRNAs binding to MEG3. TargetScan, an online tool for the prediction of miRNAs binding to CYB5R2. (i-k) The expression levels of miR-494-3p, miR-6720-5p and miR-6512-3p in blood samples from DR, D and HC patients. DR, diabetic retinopathy. D, diabetes. HC, healthy control. *P < 0.05; **P < 0.001

GSE53257 [27] from GEO DataSets was the mRNA expression profile used for six DR and five non-DR samples. With an adjusted P value < 0.05, the differentially expressed genes (DEGs) were screened from GSE53257 (Figure 1(a)). After the criteria of the log fold change (logFC) < −1, only six downregulated DEGs were identified in DR samples (Table 3). We then detected these downregulated DEGs in the blood samples and found that CYB5R2 had the lowest expression in the blood samples of DR patients (Figure 1(b-g)). The lncRNA MEG3 has been reported to play a negative role in DR progression [12,28,29], but the mechanism of MEG3 on neovascularization in DR has not been thoroughly explored. Therefore, starBase was used to predict the miRNAs binding to MEG2, and TargetScan was used to predict the miRNAs binding to CYB5R2. Using Venny 2.1.0, miR-494-3p, miR-6720-5p, and miR-6512-3p were found to be the most common miRNAs that bind to MEG3 and CYB5R2 (Figure 1(h)). The qRT-PCR results showed that miR-6512-3p and miR-6720-5p were overexpressed, in contrast to HC, in blood samples with DR, while miR-494-3p showed no change (Figure 1(i-k)). In addition, miR-6720-5p was more upregulated in DR patients than the other miRNAs. Therefore, the MEG3/miR-6720-5p/CYB5R2 axis was identified as the key regulator of DR.

**Table 3.** The downregulated DEGs in GSE53257

adj.P.Val	logFC	Gene.Symbol
0.000397	−3.08109	ACSM3
0.00386	−1.79671	DSCC1
0.010101	−1.38822	SARDH
0.010101	−1.31667	VISA
0.032456	−1.53842	CYB5R2
0.038058	−1.33973	EHHADH

### Silencing MEG3 and the promotion of neovascularization

**Figure 2.**
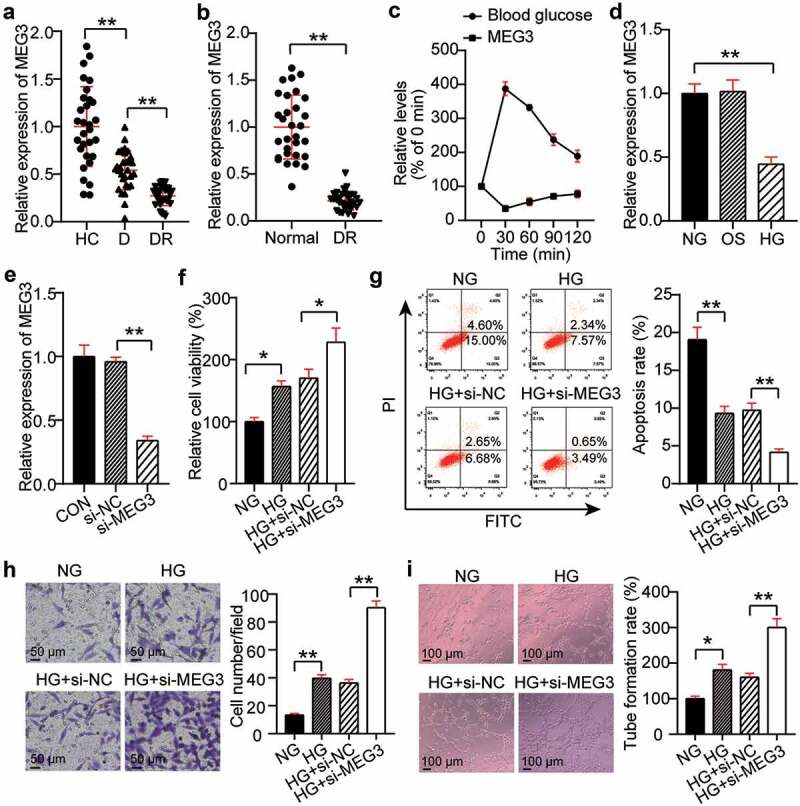
Silencing MEG3 promotes neovascularization. (a) Serum levels of MEG3 in DR, D, HC groups. (b) The expression level of MEG3 in the retinal tissue microenvironment of DR and normal rat. (c)The expression level of MEG3 in the acute fluctuation of blood glucose level. (d) The expression level of MEG3 was measured by qRT-PCR in hRMECs treated with HG, NG or OS. (e) The expression level of MEG3 was detected by qRT-PCR in hRMECs transfected with si-MEG3. (f) Cell viability was detected by using MTT assay in hRMECs transfected with si-MEG3. (g) Cell apoptosis rate was detected by using flow cytometry assay in hRMECs transfected with si-MEG3. (h) Cell migration was assessed using transwell assay after treatment with si-MEG3. Representative images were shown. (i) Tube formation assay was used to detect tubulogenesis of hRMECs transfected with si-MEG3. Representative images were shown. DR, diabetic retinopathy. D, diabetes. HC, healthy control. HG, high glucose. OS, osmotic control. NG, normal glucose. *P < 0.05; **P < 0.001

The differential expression of MEG3 in the DR, NDR, and HC groups was evaluated by qRT-PCR. Compared with the HC group, MEG3 expression in the DR and D groups decreased by 70% and 40%, respectively (Figure 2(a)). In addition, Pearson’s analysis was used to analyze the correlation between MEG3 levels and various indicators. The results showed that MEG3 expression was negatively correlated with disease course, HbA1c, FPG, TNF-α, and VEGF; but was not significantly correlated with sex, age, BMI, FBG, TC, HDL-C, LDL-C, TG, BUN, FIns, Cr, IL-1, and IL-6 (Table 4). Furthermore, the DR model of rats was established, and the expression level of MEG3 in the retinal tissue microenvironment was detected. The MEG3 level in the DR group was lower than that in the normal group (Figure 2(b)). Glucose was used to stimulate the blood glucose levels, and the association between acute fluctuations in glucose levels in the rats and MEG3 expression was analyzed. The data showed that MEG3 expression decreased as blood glucose levels increased (Figure 2(c)). Therefore, MEG3 could be DR-related. hRMECs were treated with a high concentration of glucose to establish a DR in vitro model. There was no significant difference in MEG3 levels between the OS and NG groups, while MEG3 levels were reduced by 55% in the HG group compared to the OS group (Figure 2(d)). This result indicates that the change in MEG3 does not require high osmotic pressure and is related to HG. The MEG3 level in the si-MEG3 group was 30% of that in the si-NC group (Figure 2(e)). Next, the effect of HG, or knockdown of MEG3, on neovascularization in hRMECs was examined. The MTT assay showed that the cell viability of the HG group was 1.6-fold higher than that of the NG group, and that of the HG + si-MEG3 group was 1.5 times higher than that of the HG + si-NC group (Figure 2(f)). Flow cytometry data revealed that HG treatment reduced the apoptosis rate of cells, and MEG3 knockdown further reduced the apoptosis of hRMECs (Figure 2(g)). The transwell assay revealed a 2.5-fold increase in the migration of hRMECs after HG treatment, and interference with MEG3 further upregulated the promoting effect of HG on cell migration (Figure 2(h)). In addition, the tube formation assay showed that HG treatment increased the tubulogenesis level by 1.9 times, and the low expression of MEG3 further increased the level of tubulogenesis by 1.6-fold in hRMECs treated with HG (Figure 2(i)).

**Table 4.** Pearson correlation analysis between plasma MEG3 and various indexes in the DR group

Indicators	MGE3 expression	
	r	*P*
Age (years)	0.0231	0.724
Course of disease (years)	−0.217	0.009
BMI (kg/m^2^)	−0.106	0.612
FBG (mmol/L)	0.001	0.319
HbA1c (%)	−0.584	<0.001
TC (mmol/L)	−0.019	0.943
HDL-C (mmol/L)	0.004	0.481
LDL-C (mmol/L)	−0.149	0.216
FPG (mmol/L)	−0.448	<0.001
TG (mmol/L)	−0.083	0.257
BUN (mmol/L)	−0.012	0.695
FIns (pmol/mL)	−0.057	0.472
Cr (mmol/d)	0.009	0.814
IL-1 (ng/L)	−0.196	0.271
IL-6 (ng/L)	0.045	0.793
TNF-α (ng/L)	−0.295	0.005
VEGF (ng/L)	−0.412	0.011

### MEG3 effects on neovascularization through miR-6720-5p regulation

**Figure 3.**
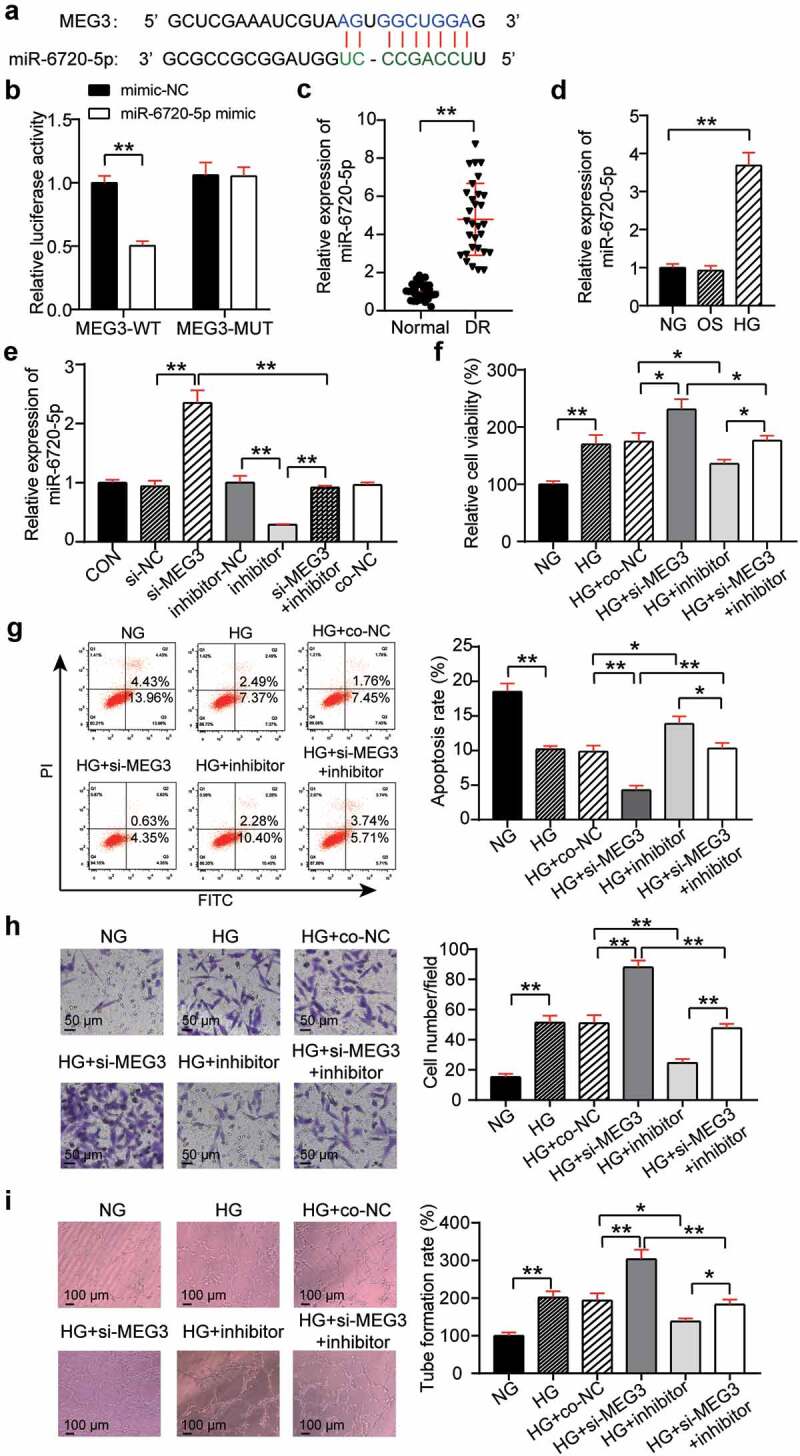
MEG3 acts on neovascularization by regulating miR-6720-5p. (a) Alignment of potential miR-6720-5p binding sites in MEG3. (b) Luciferase activity was detected in hRMECs transfected with constructs containing wild-type of MEG3 or mutated MEG3 plasmid in response to the transfection of miR-6720-5p mimic. (c) The expression level of miR-6720-5p in the retinal tissue microenvironment of DR and normal rat. (d) The expression level of miR-6720-5p was measured by qRT-PCR in hRMECs treated with HG, NG or OS. (e) The expression level of miR-6720-5p was detected by qRT-PCR in hRMECs transfected with si-MEG3 or miR-6720-5p inhibitor. (f) Cell viability was detected by using MTT assay in hRMECs transfected with si-MEG3 or miR-6720-5p inhibitor. (g) Cell apoptosis rate was detected by using flow cytometry assay in hRMECs transfected with si-MEG3 or miR-6720-5p inhibitor. (h) Cell migration was assessed using transwell assay after treatment with si-MEG3 or miR-6720-5p inhibitor. Representative images were shown. (i) Tube formation assay was used to detect tubulogenesis of hRMECs transfected with si-MEG3 or miR-6720-5p inhibitor. Representative images were shown. HG, high glucose. OS, osmotic control. NG, normal glucose. *P < 0.05; **P < 0.001

StarBase found that miR-6720-5p contained binding sites for MEG3 (Figure 3(a)). The binding relationship between miR-6720-5p and MEG3 was determined using a luciferase assay. The results showed that transfection of MEG3-WT and miR-6720-5p mimic reduced luciferase activity by 50%, while transfection of MEG3-MUT and miR-6720-5p mimic had no significant effect on luciferase activity (Figure 3(b)). This indicates that miR-6720-5p binds to MEG3. In addition, qRT-PCR analysis of retinal tissues of DR rats showed that the miR-6720-5p level in the DR group was increased compared with that in the normal group (Figure 3(c)). Analysis at the cellular level showed a 3.5-fold increase in miR-6720-5p expression in the HG group compared with that in the NG group (Figure 3(d)). This suggests that miR-6720-5p might have the opposite effect of MEG3 in DR. To confirm this hypothesis, the expression of miR-6720-5p and MEG3 was abnormally regulated in hRMECs. QRT-PCR analysis showed that silencing MEG3 increased the expression of miR-6720-5p by 2.3 times, while interfering with miR-6720-5p reduced its expression by 80% and reversed the effect of MEG3 silencing. In addition, miR-6720-5p levels in si-NC, inhibitor-NC, and co-NC were similar to those in the healthy control group (Figure 3(e)). Therefore, the co-NC group co-transfected with si-NC and inhibitor-NC was used as the co-control group treated with si-MEG3 and the miR-6720-5p inhibitor. Functionally, MTT analysis revealed that cell viability was reduced by approximately 40% in the HG + inhibitor group when compared to the HG + co-NC or si-MEG3 inhibitor groups (Figure 3(f)). Moreover, flow cytometry analysis showed that the knockdown of miR-6720-5p promoted the apoptosis of hRMECs and partially eliminated the inhibition of apoptosis caused by MEG3 downregulation (Figure 3(g)). Furthermore, interference with miR-6720-5p reduced hRMEC migration by 60% and reversed the migration promotion effect of the silenced MEG3 gene (Figure 3(h)). Additionally, after the knockdown of miR-6720-5p, the level of tubulogenesis was inhibited by approximately 40%, and the effect of MEG3 knockdown on tubulogenesis was partially eliminated (Figure 3(i)).

### CYB5R2 is the target gene of miR-6720-5p, and knockdown of CYB5R2 reversed the inhibitory effect of the miR-6720-5p inhibitor on neovascularization

**Figure 4.**
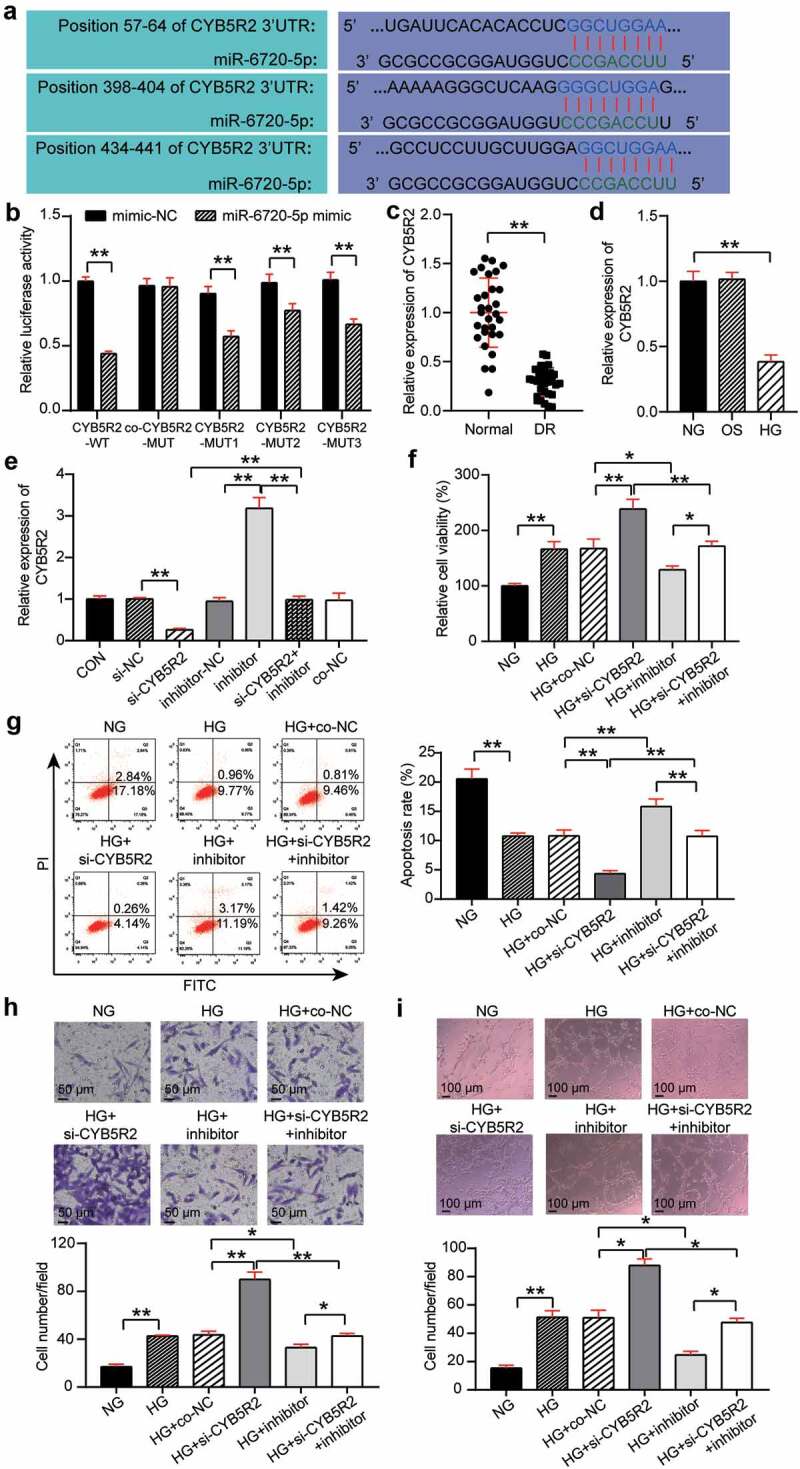
CYB5R2 is the target gene of miR-6720-5p, and knockdown of CYB5R2 reversed the inhibitory effect of miR-6720-5p inhibitor on neovascularization. (a) Alignment of potential CYB5R2 binding sites in miR-6720-5p. (b) Luciferase activity was detected in hRMECs transfected with constructs containing wild-type of CYB5R2 or mutated CYB5R2 plasmid in response to the transfection of miR-6720-5p mimic. (c) The expression level of CYB5R2 in the retinal tissue microenvironment of DR and normal rat. (d) The expression level of CYB5R2 was measured by qRT-PCR in hRMECs treated with HG, NG or OS. (e) The expression level of CYB5R2 was detected by qRT-PCR in hRMECs transfected with si-CYB5R2 or miR-6720-5p inhibitor. (f) Cell viability was detected by using MTT assay in hRMECs transfected with si-CYB5R2 or miR-6720-5p inhibitor. (g) Cell apoptosis rate was detected by using flow cytometry assay in hRMECs transfected with si-CYB5R2 or miR-6720-5p inhibitor. (h) Cell migration was assessed using transwell assay after treatment with si-CYB5R2 or miR-6720-5p inhibitor. Representative images were shown. (i) Tube formation assay was used to detect tubulogenesis of hRMECs transfected with si-CYB5R2 or miR-6720-5p inhibitor. Representative images were shown. HG, high glucose. OS, osmotic control. NG, normal glucose. *P < 0.05; **P < 0.001

TargetScan showed that CYB5R2 contained three binding sites for miR-6720-5p (Figure 4(a)). Three binding sites were mutated to obtain variants of CYB5R2(MUT1, MUT2, MUT3, and Co-MUT), which were co-transfected with miR-6720-5p mimic into hRMECs, and the results showed that the luciferase activity decreased by 40%, 20%, and 30%, respectively, after a single site mutation. The luciferase activity did not significantly change after all three sites were mutated. In addition, the luciferase activity of CYB5R2-WT was reduced by 60%. Therefore, miR-6720-5p targets CYB5R2 (Figure 4(b)). Moreover, the analysis of retinal tissue of DR rats revealed that the level of CYB5R2 in this group was lower than that in the normal group (Figure 4(c)). In addition, the expression of CYB5R2 decreased by 60% after HG treatment compared with that in the OS group (Figure 4(d)). In the hRMECs, CYB5R2 expression increased by 3.2 times after the miR-6720-5p inhibitor treatment, and decreased by 80% after si-CYB5R2 treatment, which, as a result, eliminated the effect of miR-6720-5p inhibitor (Figure 4(e)). The MTT assay showed that the viability of HG-induced hRMECs increased by 1.3-fold after CYB5R2 silencing, and the pro-proliferation effect of the miR-6720-5p inhibitor treatment was reversed (Figure 4(f)). Additionally, knockdown of CYB5R2 reduced the apoptosis rate of hRMECs and reversed the pro-apoptotic effect of the miR-6720-5p inhibitor (Figure 4(g)). Moreover, cell migration in the HG si-CYB5R2 group was enhanced by approximately 1.7 times compared to the HG and co-NC, and si-CYB5R2 inhibitor groups (Figure 4(h)). Moreover, the inhibition of CYB5R2 increased tubulogenesis by a factor of 1.5, and partially eliminated the downregulating effect of miR-6720-5p on tubulogenesis (Figure 4(i)).

## Discussion

Vascular changes in DR are related to cell damage and pathological changes in the blood retinal barrier capillaries [30]. Based on the current evidence, when DR progresses, capillary perfusion may be impaired, leading to retinal ischemia, followed by the upregulation of pro-angiogenic factors, thus leading to pathologic neovascularization [31]. Current treatment strategies for DR aim to control microvascular complications. These strategies include intravitreous drugs, laser photocoagulation, and vitreous surgery [32]. Although considerable progress has been made in understanding the key disease factors associated with DR, current treatments have not significantly improved visual acuity [33]. Therefore, there is an urgent need to explore the mechanism of DR neovascularization and evaluate new pharmacological therapies.

As a tumor suppressor gene, MEG3 inhibits the occurrence and development of tumors by inhibiting the proliferation, migration, and apoptosis of cancer cells [34]. LncRNA-MEG3 has been shown to have some effect on diabetes-related microvascular dysfunction [35]. Consistent with the results of Zhang et al. [12], in this study we found a low level of MEG3 in the plasma of DR patients and retinal tissues of DR rats. In addition, Xiao et al. [36] found that levels of MEG3 decreased in hRMECs induced by HG, and that MEG3 inhibited HG-induced apoptosis and inflammatory responses. Based on these findings, this study explored whether the knockdown of MEG3 further aggravated hRMEC proliferation and migration levels and angiogenesis, and whether it inhibited apoptosis. Previous studies have revealed that MEG3 may play a role in treating DR neovascularization, to a certain extent.

LncRNAs may regulate the expression of target mRNAs by functioning as ceRNAs or miRNA sponges [37]. The lncRNA-miRNA-mRNA network has been extensively studied as biomarker and potential therapeutic target for disease diagnosis and prognosis [38]. For example, Wan et al. [39] established multiple ceRNA networks in patients with diabetic vascular complications. Tong et al. [40] revealed that MEG3 inhibited the NF-κB signaling pathway by targeting the miR-34a/SIRT1 axis; thus reducing HG-induced cell apoptosis and inflammatory responses. LncRNAs usually exert their biological functions by sponging miRNAs [41]. Through bioinformatic analysis, we found that miR-6720-5p may be a target of MEG3. Further, the luciferase assay verified that miR-6720-5p was sponged by MEG3 in hRMECs. In addition, functional analysis showed that interference with miR-6720-5p inhibited the proliferation and migration of hRMECs and angiogenesis. This indicates that in DR, miR-6720-5p plays an opposite role to MEG3. Furthermore, the rescue analysis showed that interference with miR-6720-5p reversed the effect of MEG3 knockdown on hRMEC function, suggesting that the effect of MEG3 on DR may be achieved through its sponging of miR-6720-5p.

Several studies have shown that CYB5R2 is under expressed in human DR [18]. In addition, Ming et al. [17] found that CYB5R2 is involved in the regulation of angiogenesis. In this study, CYB5R2 was also shown to be downregulated in the serum of patients with DR. Functionally, the knockdown of CYB5R2 promoted proliferation, migration, and angiogenesis, while inhibited apoptosis of hRMECs. These results indicate that CYB5R2 has value in treating DR. Additionally, target analysis revealed for the first time that CYB5R2 was the target gene of miR-6720-5p, and reversed the effect of miR-6720-5p on hRMECs. MEG3 acts as a ceRNA to relieve neovascularization in DR through sponging and targeting the miR-6720-5p of CYB5R2.

VEGF is an important angiogenic factor in DR, and new evidence suggests that the VEGF antibody treatment may be a new therapy for DR [42,43]. Multiple analyses have also shown that both MEG3 and CYB5R2 have a regulatory effect on VEGF [12,17]. Therefore, future studies will focus on the role of VEGF in DR mediated by MEG3/miR-6720-5p/CYB5R2. In addition, elucidating the effect of MEG3/miR-6720-5p/CYB5R2 on DR neovascularization at the cellular level is not sufficient to fully understand the underlying mechanism. Therefore, the expression of these genes in the retinal tissue microenvironment of DR patients needs to be further explored in the future.

## Conclusion

In summary, this study showed that MEG3 and CYB5R2 were downregulated in DR, while miR-6720-5p was downregulated. It was found for the first time that silencing MEG3 downregulates the expression of CYB5R2 by acting as a sponge for miR-6720-5p, and exerts a positive regulatory effect, thereby promoting angiogenesis and the proliferation and migration of hRMECs, and inhibiting apoptosis. This study provides new insights into the treatment of DR, which may contribute to the development of neovascularization in DR-targeted therapy.

## Data Availability

The datasets used and/or analyzed during the current study are available from the corresponding author on reasonable request.

## Highlights

- MEG3/miR-6720-5p/CYB5R2 axis is associated with DR.
- Silencing MEG3 promotes neovascularization.
- MEG3 acts on neovascularization by regulating miR-6720-5p.
- CYB5R2 is the target gene of miR-6720-5p, and knockdown of CYB5R2 reversed the inhibitory effect of the miR-6720-5p inhibitor on neovascularization.
